# A Recombinant *Potato virus Y* Infectious Clone Tagged with the Rosea1 Visual Marker (PVY-Ros1) Facilitates the Analysis of Viral Infectivity and Allows the Production of Large Amounts of Anthocyanins in Plants

**DOI:** 10.3389/fmicb.2017.00611

**Published:** 2017-04-06

**Authors:** Teresa Cordero, Mohamed A. Mohamed, Juan-José López-Moya, José-Antonio Daròs

**Affiliations:** ^1^Instituto de Biología Molecular y Celular de Plantas (Consejo Superior de Investigaciones Científicas – Universidad Politécnica de Valencia)Valencia, Spain; ^2^Centre for Research in Agricultural Genomics, Consejo Superior de Investigaciones Científicas – Institut de Recerca i Tecnologia Agroalimentaries – Universitat Autònoma de Barcelona – Universitat de BarcelonaBarcelona, Spain

**Keywords:** potato virus Y, anthocyanin, silver nanoparticles, aphid vector, molecular farming

## Abstract

*Potato virus Y* (PVY) is a major threat to the cultivation of potato and other solanaceous plants. By inserting a cDNA coding for the *Antirrhinum majus* Rosea1 transcription factor into a PVY infectious clone, we created a biotechnological tool (PVY-Ros1) that allows infection by this relevant plant virus to be tracked by the naked eye with no need for complex instrumentation. Rosea1 is an MYB-type transcription factor whose expression activates the biosynthesis of anthocyanin pigments in a dose-specific and cell-autonomous manner. Our experiments showed that the mechanical inoculation of solanaceous plants with PVY-Ros1 induced the formation of red infection foci in inoculated tissue and solid dark red pigmentation in systemically infected tissue, which allows disease progression to be easily monitored. By using silver nanoparticles, a nanomaterial with exciting antimicrobial properties, we proved the benefits of PVY-Ros1 to analyze novel antiviral treatments in plants. PVY-Ros1 was also helpful for visually monitoring the virus transmission process by an aphid vector. Most importantly, the anthocyanin analysis of infected tobacco tissues demonstrated that PVY-Ros1 is an excellent biotechnological tool for molecular farming because it induces the accumulation of larger amounts of anthocyanins, antioxidant compounds of nutritional, pharmaceutical and industrial interest, than those that naturally accumulate in some fruits and vegetables well known for their high anthocyanin content. Hence these results support the notion that the virus-mediated expression of regulatory factors and enzymes in plants facilitates easy quick plant metabolism engineering.

## Introduction

Tagging plant virus clones with marker genes to track infection has considerably contributed to advance in plant virology in recent decades (Tilsner and Oparka, 2010). Pioneering works used tags to code for enzymes, e.g., acetyl chloramphenicol transferase or luciferase, which facilitated the quantification of virus replication (French et al., 1986; Joshi et al., 1990). The construction of an infectious clone of *Tobacco etch virus*, which harbored a heterologous cDNA that encoded bacterial enzyme beta-glucuronidase (GUS), was a step further because this recombinant clone permitted the cells and tissues where the virus expressed and replicated its genome to be visualized (Dolja et al., 1992). However, GUS activity had to be revealed by infiltrating plant tissues with a suitable chromogenic substrate. The incorporation of a new generation of markers, which encoded fluorescent proteins, definitively boosted this strategy to study plant viruses (Baulcombe et al., 1995). Unlike GUS, fluorescent proteins, like *Aequorea victoria* green fluorescent protein (GFP), do not need a substrate to be revealed (Chalfie et al., 1994; Rodriguez et al., 2016). We more recently introduced a new type of marker gene that allowed the visual monitoring of infected tissues by the naked eye without having to resort to complex instruments (Bedoya et al., 2012). Virus-mediated expression of snapdragon (*Antirrhinum majus* L.) MYB-type transcription factor Rosea1 induces the accumulation of reddish anthocyanins exclusively in tissues infected by the virus (Bedoya et al., 2012). A triad of transcription factors, namely an MYB, a bHLH and a WD40, form a ternary complex that activates the expression of anthocyanin biosynthetic genes (Zhang et al., 2014; Passeri et al., 2016). The MYB transcription factor is the limiting element of this complex (Allan et al., 2008), which explains why virus-mediated Rosea1 expression closely correlates with the accumulation of anthocyanins, which is proportional to viral load (Bedoya et al., 2012). However, this technology is not universal and depends on factors like endogenous virus capacity to express foreign proteins or the affinity of *A. majus* Rosea1 to interact with companion endogenous transcription factors (Majer et al., 2017). Thus its applicability to a particular plant virus-host system must be tested.

**FIGURE 1:**
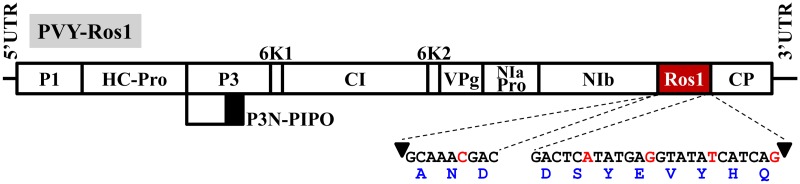
**Schematic representation of the PVY recombinant clone in which transcription factor Rosea1 was expressed between NIb and CP: PVY-Ros1**. Lines represent the viral 5′ and 3′ untranslated regions (UTR), and boxes denote viral cistrons P1, HC-Pro, P3, P3N-PIPO, 6K1, CI, 6K2, VPg, NIaPro, NIb and CP, as indicated. Rosea1 (Ros1) is indicated by a red box. The nucleotide and amino acid sequences, added to both ends of *Rosea1* to complement the split NIb/CP proteolytic site, are also indicated. Arrowheads indicate the cleavage site. The nucleotides in red correspond to silent mutations to avoid homologous repetitions in the PVY-Ros1sequence.

*Potato virus Y* (PVY) is currently considered the major viral threat to potato (*Solanum tuberosum* L.) cultivation (Karasev and Gray, 2013; Quenouille et al., 2013), and is ranked number 5 in the top 10 plant viruses in molecular plant pathology (Scholthof et al., 2011). This virus is the type member of the very large genus *Potyvirus*, and the family *Potyviridae*, of plus-strand RNA viruses (Gibbs and Ohshima, 2010). It has a wide host range that includes plants in nine botanical families, although economically important diseases concentrated in cultivated solanaceous plants like potato, tomato (*S. lycopersicum* L.), pepper (*Capsicum* spp.), tobacco (*Nicotiana* spp.), or petunia (*Petunia* spp.) (Quenouille et al., 2013). PVY is transmitted by more than 40 aphid (family *Aphididae*) species in a non-persistent manner. Transmission by worldwide distributed aphid *Myzus persicae* Sulz. is particularly efficient. Phylogenetic and epidemiologic analyses indicate that PVY originated in the Americas, but the virus is currently distributed worldwide (Quenouille et al., 2013). As it is typical of most potyviruses (genus *Potyvirus*), the PVY genome consists of an almost 10000 nucleotide (nt) long RNA molecule that is covalently linked at the 5′ end to a viral protein genome-linked (VPg), and contains a poly(A) tail at the 3′ end. This genomic RNA is encapsidated by approximately 2000 copies of a single coat protein (CP) in elongated and flexuous virions. The viral genome encodes a large polyprotein and viral products (P1, HC-Pro, P3, 6K1, CI, 6K2, VPg, NIaPro, NIb, and CP; **Figure 1**) result from a regulated cascade of proteolytic processing (Revers and García, 2015). An additional product (P3N-PIPO) results from a frameshift at the P3 cistron, recently shown to be generated through a polymerase slippage mechanism (Olspert et al., 2015; Rodamilans et al., 2015).

We wondered whether the Rosea1 visual marker could be applied to such a relevant virus as PVY, and, particularly whether the marker confers any advantage to either the aphid transmission analysis or the effect of antiviral treatments, among others. To this end, we built an infectious clone of a wild isolate of PVY tagged with *Rosea1* (PVY-Ros1). We observed that this recombinant clone induced red spots in mechanically inoculated tissue, which is indicative of infection foci, and solid dark red coloration on upper non-inoculated leaves when systemic infection was established. Using silver nanoparticles, which are an easy-to-produce novel nanomaterial with exciting antimicrobial activities, we proved that PVY-Ros1 allows the very precise quantification of the effect of antiviral treatments in plants. Interestingly, aphids that fed on red-colored tissue efficiently transmitted the disease during a process that can be easily tracked thanks induction of discrete visible infection foci, which presumably surround the aphid puncture site during inoculation. Finally, we observed that PVY-Ros1 can be used in molecular farming to very quickly induce the production of large amounts of anthocyanins, antioxidant compounds with nutritional, pharmaceutical and industrial applications, in biofactory crops.

## Materials and Methods

### Construction of a PVY Recombinant Clone Tagged with *Rosea1*

RNA was purified using silica gel spin columns (Zymo Research) from potato leaf tissue infected with a wild Canadian isolate of PVY (RB strain). With this RNA preparation, three different cDNAs that covered the whole viral genome were synthesized using RevertAid reverse transcriptase (Thermo Scientific) and oligodeoxynucleotide primers PI to PIII (the sequences of all the primers used in this work are indicated in Supplementary Table S1). Each cDNA served as a template to amplify the PVY genome in three different fragments (5′, central and 3′) by the polymerase chain reaction (PCR) using Phusion high-fidelity DNA polymerase (Thermo Scientific) and primers PIV and PV (5′ fragment), PVI and PVII (central fragment) and PVIII and PIX (3′ fragment). These primers were flanked with the recognition site of type-IIS restriction enzyme *Eco*31I (Supplementary Table S1). Amplification products were cloned at the *Eco*RV site of pBSΔE, a derivative of pBluescript II KS+ (GenBank accession number X52327.1), at which an *Eco*31I recognition site was mutagenized. A selected recombinant plasmid, which contained the central part of the PVY genome, was used as a template to mutagenize an endogenous *Eco*31I recognition site by PCR with primers PX and PXI by introducing a silent mutation. Another selected plasmid that contained the 3′ fragment of the PVY genome was used as a template to insert a cDNA coding for the *A. majus* Rosea1 transcription factor (GenBank accession number DQ275529.1) between cistrons NIb and CP by PCR amplification with Phusion DNA polymerase, digestion with *Eco*31I (Thermo Scientific) and ligation with T4 DNA ligase (Thermo Scientific). The plasmid was opened by PCR using primers PXII and PXIII. *Rosea1* cDNA was amplified with primers PXIV and PXV, and was flanked by extra sequences that encoded amino acids to complement the split NIb/CP NIaPro cleavage site (**Figure 1**). Finally, the cDNAs that corresponded to the 5′ and central fragments of the PVY genome, as well as the 3′ fragment with the *Rosea1* insertion, were released from their respective plasmids using *Eco*31I. They were assembled (Engler and Marillonnet, 2014) in binary plasmid pG35Z (Cordero et al., 2017) by ligation with T4 DNA ligase. We named the resulting plasmid pGPVY-Ros1.

### Plant Inoculation

For plant agroinoculation, strain C58C1 of *Agrobacterium tumefaciens*, which harbored helper plasmid pCLEAN-S48 (Thole et al., 2007), was electroporated with pGPVY-Ros1. Transformed *A. tumefaciens* clones were first selected in plates with 50 μg/ml of kanamycin, 7.5 μg/ml of tetracycline and 50 μg/ml of rifampicin. The selected clones were further grown in liquid media at 28°C with only 50 μg/ml of kanamycin and were used to agroinoculate 4-week-old *Nicotiana benthamiana* Domin plants in two leaves (Bedoya and Daròs, 2010). Liquid cultures of the transformed *A. tumefaciens* were grown to optical density at 600 nm (OD_600_) of approximately 1.0. Cells were recovered by centrifugation and were resuspended at an OD_600_ of 0.5 in 10 mM MES-NaOH, pH 5.6, 10 mM MgCl_2_, and 150 μM acetosyringone. Cultures were induced for 3 h at 28°C and were used to agroinoculate *N. benthamiana* plants. For the mechanical inoculation of plants, crude extracts of infected tissues, obtained in 20 volumes of 50 mM of potassium phosphate, pH 8.0, 1% polyvinylpyrrolidone 10, 1% polyethylene glycol 6000 and 10 mM of 2-mercaptoethanol, were rubbed on the leaf surface in the presence of carborundum using a cotton swab (Bedoya and Daròs, 2010). Five-week-old tobacco (*N. tabacum* L., cv. Xanthi nc), 4-week-old tomato (*S. lycopersicum* L., cv. Marglobe) and 6-week-old potato (traditional cultivar from Soria, Spain) plants were used for mechanical inoculation. In all cases, plants were kept in a growth chamber at 25°C under a 12-h day-night photoperiod. For aphid transmission, standard plant-to-plant transmission experiments were performed, as previously described (Atreya et al., 1995). Apterae individuals from a colony of *M. persicae*, kindly provided by A. Fereres (ICA-CSIC, Madrid, Spain), and reared in the laboratory on tobacco plants, were collected and kept in glass vials for a 2-h fasting period. Aphids were allowed a 10-min acquisition access period on the PVY-Ros1-infected leaves, and were then transferred to the test plants, either individually or in groups of 5 or 10. After an overnight inoculation access period (14–18 h), aphids were killed by spraying with insecticide, and plants were placed in a growth chamber for symptoms to develop.

### PVY Diagnosis by RT-PCR

The RNA preparations obtained from leaf tissue using silica gel spin columns were reverse-transcribed with RevertAid reverse transcriptase and primer PIII. The reverse transcription products were PCR-amplified with *Thermus thermophilus* DNA polymerase (Biotools), using primers PXVI and PXVII to amplify the PVY CP cistron (801 bp), and were revealed by gel electrophoresis (1% agarose) and ethidium bromide staining.

### Synthesis of Silver Nanoparticles and Treatment of Plants

Endophytic fungus *Curvularia lunata* was aerobically grown in potato dextrose broth at 26°C for 3 days with agitation. The fungal mat was washed with sterile water and an aliquot of 10 g was further agitated in 100 ml of water for 2 days at 26°C. The culture exudate was recovered by filtration with Whatman number 1 paper and was mixed with 100 ml of 1 mM AgNO_3_. The mixture was then incubated in the dark at room temperature until color changed. The silver reduction reaction and the formation of silver nanoparticles were monitored for approximately 1 week by a spectrophotometric analysis. The resulting silver nanoparticles were purified, dried and characterized by spectroscopic and microscopic analyses, as previously described (Abdel-Hafez et al., 2016). A fine dispersion of silver nanoparticles in water at 1000 parts per million (ppm) was obtained by three cycles of 5-min stirring and 5-min sonication. From this dispersion, a 1:10 dilution in water (100 ppm) was prepared. These silver nanoparticle preparations were sprayed on 5-week-old tobacco (cv. Xanthi nc) plants. Two days later, the true leaves 5 and 6 of these plants were mechanically inoculated with PVY-Ros1 in the presence of carborundum, as described above.

### Analysis of Anthocyanins

The tissue samples from the systemic leaves from tobacco plants were collected at 12 days post-inoculation (dpi), unless indicated, and frozen at -80°C. While frozen, these tissue samples were mechanically ground and mixed. For each sample, a representative aliquot of approximately 1 g of tissue was homogenized in 10 volumes of extraction solution (1% HCl in methanol) with a Polytron (Kinematica). Extracts were vigorously vortexed, incubated on ice for 1 h with sporadic vortexing, and finally clarified by centrifugation for 10 min at 12000 g. Supernatants were diluted to 1:10 in extraction solution (final ratio tissue:extraction solution 1:100) and analyzed spectrophotometrically (UV-3100PC, VWR). Anthocyanin identification was done in a UPLC/PDA/qTOF-MS instrument (micromass Q-TOF micro, Waters). Separation was performed in an ACQUITY BEH C18 column (150 × 2.1 mm i.d., 1.7 μm) with a mobile phase that consisted of formic acid:water (1:1000 v/v; phase A) and formic acid:acetonitrile (1:1000 v/v; phase B). The gradient conditions were: 95% A for 5 min, 95 to 90% A in 14 min, 90 to 80% A in 15 min, 80 to 65% A in 10 min, 65 to 30% A in 5 min, 30 to 0% A in 1 min, held at 100% B for 5 min, returned to 95% A in 1 min, and equilibrated for 4 min before the next injection. The flow rate was 0.4 ml/min and the sample injection volume was 5 μl. Column and sample temperatures were 40 and 10°C, respectively. UV-visible spectra were acquired within the wavelength range of 220 to 800 nm with 1.2-nm resolution and a 20 points/s sampling rate. The MS analysis was performed by electrospray ionization in the negative mode. The mass spectrometry conditions were as follows: capillary voltage 2900 V, cone voltage 30 V; desolvation temperature 300°C, source temperature 120°C, cone gas flow 50 l/h, desolvation gas flow 450 l/h. MS data were acquired in the centroid mode within an m/z range of 100–1500 in a scan time of 0.5 s and an interscan time of 0.1 s. MS was calibrated using sodium formate, and leu-enkephalin was used as the lock mass with a LockSpray exact mass ionization source. MassLynx version 4.1 and Q-tof Micro version 4.1 (Waters) were used to control instruments and to calculate accurate masses.

## Results and Discussion

### A Recombinant PVY Clone Tagged with Rosea1 Is Infectious and Induces a Dark Red Pigmentation of Infected Tissue

To analyze whether the Rosea1 marker is operational to visually reveal PVY infected tissues, starting with a wild isolate of this virus, we constructed a full-length clone in which a cDNA coding for *A. majus Rosea1* was inserted between the cistrons NIb and CP (PVY-Ros1, **Figure 1**). *Rosea1* cDNA was flanked by artificial sequences that code for amino acids to complement both sides of the split NIb/CP proteolytic site. At the protein level, these sequences regenerate the original NIb/CP cleavage site at both sides, but at the nucleotide level they include silent mutations to avoid sequence repetitions, which could facilitate marker deletion by homologous recombination during viral replication (**Figure 1**). The recombinant PVY-Ros1 clone was fully sequenced (Supplementary Datasheet S1) and deposited in GenBank (accession number KY780083). Apart from *Rosea1* and the sequences to complement proteolysis, a BLAST search indicated that cloned PVY mostly resembled a mild Canadian isolate of PVY (sequence variant RB of PVY-O isolate, GenBank accession number HM367076) (Nie et al., 2011). The sequences were 99.9% identical, and only differed in one insertion (A1) and five mutations (G4550A, A4907G, G5794A, A6533C, C7280T, and A7338G; numbering corresponds to PVY-Ros1). Note that the G4550A silent mutation was introduced into the recombinant clone in order to eliminate an endogenous *Eco*31I recognition site and to facilitate manipulation. A6533C and C7280T are silent mutations, whereas A4907G, G5794A, and A7338G induce amino acid substitutions I1574M, S1870N, and T2385A with respect to HM367076.

**FIGURE 2:**
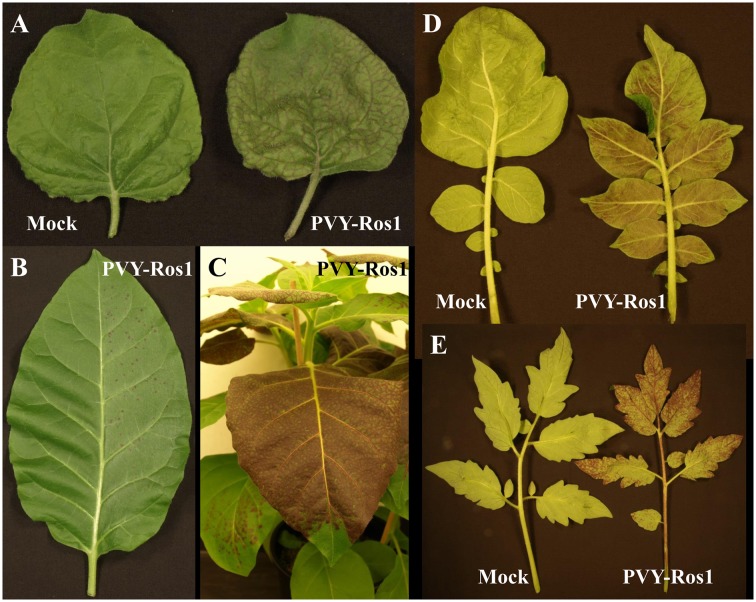
**Plants infected with PVY-Ros1 that show red pigmentation**. **(A)** Upper non-inoculated leaves from mock (left) and PVY-Ros1 (right) agroinoculated *N. benthamiana* plants showing veinal-associated anthocyanin accumulation. **(B)** *N. tabacum* leaf mechanically inoculated (right side) with PVY-Ros1. The picture was taken at 6 dpi. **(C)** Detail of an *N. tabacum* plant infected with PVY-Ros1 (11 dpi) showing solid dark red pigmentation. **(D,E)** Upper non-inoculated leaves from potato **(D)** and tomato **(E)** plants mock-inoculated (left) and mechanically inoculated with PVY-Ros1 (right). Pictures were taken at 6 and 19 dpi, respectively.

Three *N. benthamiana* plants were agroinoculated in two leaves with an *A. tumefaciens* clone, which harbored plasmid pGPVY-Ros1 that contained PVY-Ros1. One of the three agroinoculated plants showed infection symptoms at 26 dpi in upper non-inoculated leaves. Interestingly, symptomatic tissues turned red (**Figure 2A**). Considering the relatively low infection rate observed in this agroinoculation experiment, a similar set of three *N. benthamiana* plants was directly inoculated mechanically with plasmid pGPVY-Ros1. One of the three mechanically inoculated plants also displayed infection symptoms at 31 dpi, which also turned red. We used infected tissue from these plants to prepare crude extracts to mechanically inoculate tobacco (*N. tabacum*) plants. All the mechanically inoculated leaves started to show red infectious foci at 5 dpi, which were clearly visible at 6 dpi (**Figure 2B**). All the mechanically inoculated plants started to show infection symptoms on their upper non-inoculated leaves at 6 dpi, which were easily recognized thanks to the simultaneous red pigmentation. In all the inoculated plants, symptomatic tissue showed intense solid dark red coloration at 12 dpi (**Figure 2C**). When these same infectious extracts were used to mechanically inoculate potato and tomato plants, all the plants also became infected and exhibited red pigmentation in infected tissue (**Figures 2D,E**).

**FIGURE 3:**
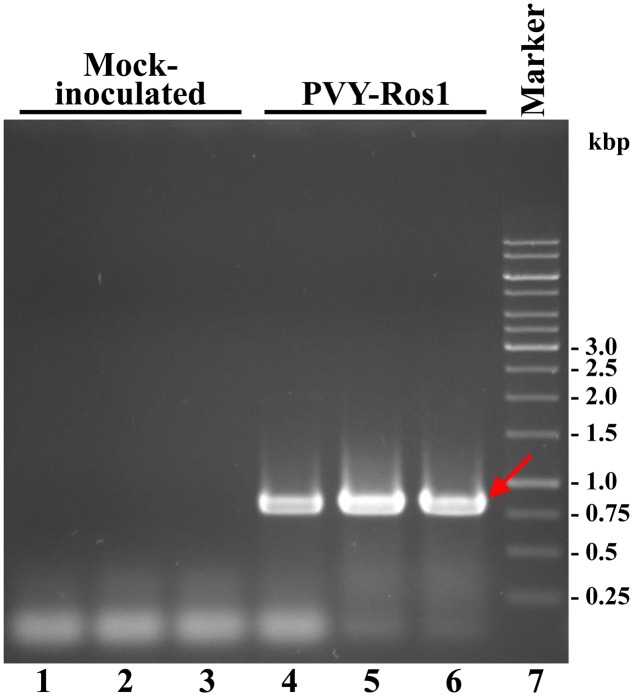
**Diagnosis of PVY-Ros1 in tobacco plants by RT-PCR**. Amplification products from the RNA preparations of three plants mock-inoculated (lanes 1 to 3), and inoculated with PVY-Ros1 (lanes 4 to 6), were separated by electrophoresis in agarose gel and revealed by ethidium bromide staining. Lane 7 contains a DNA marker with sizes in bp on the right. The red arrow points to the amplification product that corresponds to cistron PVY CP (801 bp).

An RT-PCR analysis of the tissues from the tobacco mock-inoculated controls and infected plants (8 dpi) demonstrated that red symptomatic tissues accumulated PVY-Ros1. The electrophoretic analysis of the amplification product revealed a specific band whose size matched the 801-bp size expected for the PVY cistron CP in the samples that corresponded to the PVY-Ros1-infected plants (**Figure 3**). The sequence analysis of these PCR products confirmed PVY identity.

Taken together, these results indicated that the recombinant clone PVY-Ros1 contained in plasmid pGPVY-Ros1 was infectious and efficiently expressed the *A. majus* Rosea1 transcription factor which, at the time, activated anthocyanin biosynthesis in the infected tissues of the different tested solanaceous hosts: *N. benthamiana*, *N. tabacum*, potato and tomato. However, the results also suggested that pGPVY-Ros1 was rather an unstable plasmid because, when starting with the plasmid, the infection rate obtained by either agroinoculation or direct mechanical inoculation was low. Instability of the plasmids that contain full-length clones of plant RNA viruses is frequent (Boyer and Haenni, 1994; Bedoya and Daròs, 2010). In addition to low infectivity, infection from plasmid (either by agroinoculation or mechanical inoculation) also showed an extended delay compared to the mechanical inoculation using an infectious extract. This suggests poor expression efficiency of viral full-length cDNA. As the expression in this construct was driven by the very strong *Cauliflower mosaic virus* (CaMV) 35S promoter and terminator, one possible explanation for this low infectivity could be presence of cryptic introns in the PVY sequence, which could result in undesired splicing in most transcripts. The undesired presence of cryptic introns has been previously shown in *Tobacco mosaic virus* (Marillonnet et al., 2005). Although, presence of cryptic introns has never been shown in the case of potyviruses, conversely intron insertion has been used to enhance clone stability and infectivity (Johansen, 1996; López-Moya and García, 2000). In any case, our experiments indicated the possibility of inducing infection with PVY-Ros1 by both agroinoculation and direct mechanical inoculation of pGPVY-Ros1. Most importantly, after obtaining some initial infected tissue, it could be used as a fast and efficient source of PVY-Ros1 inoculum for successive experiments. Nonetheless, infection efficiency of agroinoculated PVY-Ros1 may be improved by the co-expression of tombusvirus p19 silencing suppressor (Saxena et al., 2011). Recently, a PVY clone tagged with GFP has been proposed as a versatile tool for the functional analysis of plant-virus interaction (Rupar et al., 2015). Based on direct visual detection, PVY-Ros1 must be able to complement this tool with new experimental possibilities.

### PVY-Ros1 Facilitates the Quantitative Analysis of the Antiviral Activity of Silver Nanoparticles

**FIGURE 4:**
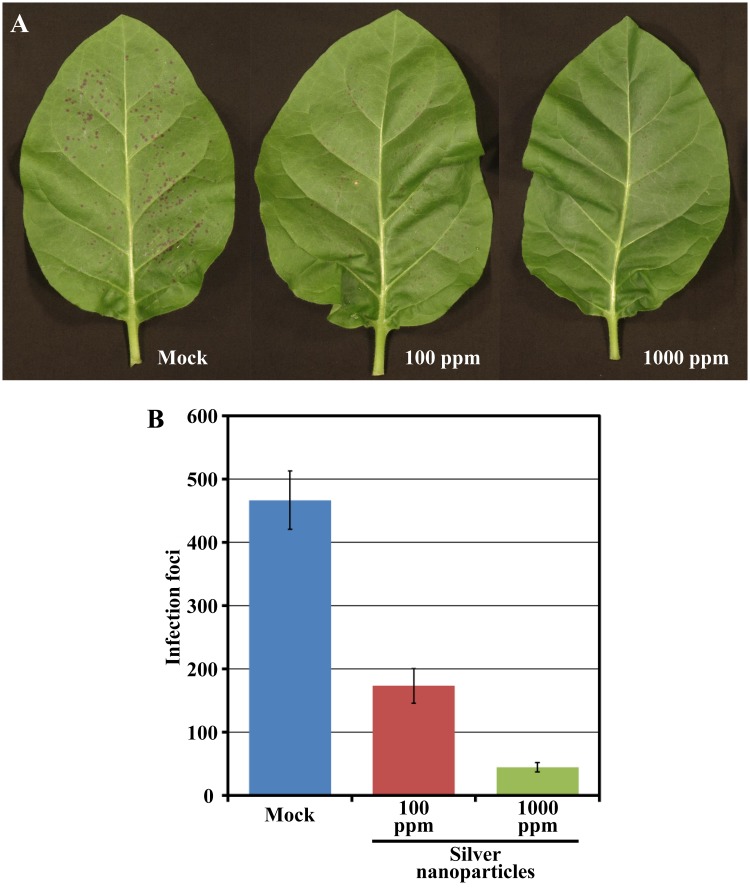
**Effect of silver nanoparticles on PVY-Ros1 infectivity**. **(A)** Pictures of representative leaves of tobacco plants mock-treated or sprayed with a preparation of silver nanoparticles in water at 100 and 1000 ppm, as indicated. Two days after treatment, leaves were mechanically inoculated with PVY-Ros1. Pictures were taken at 6 dpi with PVY-Ros1. **(B)** Histogram of the average number of infection foci per leaf of the six plants treated with silver nanoparticles, as indicated, and mechanically inoculated with PVY-Ros1 2 days later. Foci were quantified at 6 dpi with PVY-Ros1. Error bars represent the standard error median.

The speed and easiness with which PVY-Ros1-derived red spots and patterns are monitored or recorded in real time without having to resort to complex instrumentation make this recombinant virus an excellent tool to analyze the effect of environmental growth conditions, the host genetic background or phytosanitary treatments in virus infection. To acquire proof of this concept, we thought about treating tobacco plants with silver nanoparticles, a novel kind of nanomaterial for which exciting antimicrobial activities have been reported (Mishra and Singh, 2015), and then inoculating the treated tissues with PVY-Ros1. Sets of six tobacco plants (6 weeks old) were sprayed with a preparation of *C. lunata*-derived silver nanoparticles at 100 ppm and 1000 ppm in water. In this experiment, we included a set of mock-sprayed plants. Two days later, two leaves per plant were mechanically inoculated with aliquots of the same PVY-Ros1 inoculum. Red infection foci were clearly visible at 6 dpi (**Figure 4A**). The inoculated leaves were harvested and foci were counted (Supplementary Table S2). Whereas an average of 233 individual infection foci per leaf was observed on the mock inoculated leaves, only an average of 87 and 22 foci was observed on the leaves treated with 100 and 1000 ppm of silver nanoparticles, respectively (**Figure 4B**). Hence the use of PVY-Ros1 very precisely showed the protective effect of silver nanoparticles against infection by a plant RNA virus. Antiviral activity of silver nanoparticles has been repeatedly shown in human viruses (Lu et al., 2008; Lara et al., 2011) or bacteriophages (Narasimha et al., 2012), but in only one recent report in plant viruses (Elbeshehy et al., 2015). It is interesting to note that while in this report authors did not detect any beneficial effect of silver nanoparticles in pre-infection treatment and only remarkable positive results were observed in post-infection treatment (Elbeshehy et al., 2015), we clearly observed a significant beneficial effect of silver nanoparticles in treatments 48 h ahead of viral challenging thanks to the Rosea1-based easy monitoring of infection foci.

### PVY-Ros1 Enables the Visual Monitoring of Aphid Transmission

**FIGURE 5:**
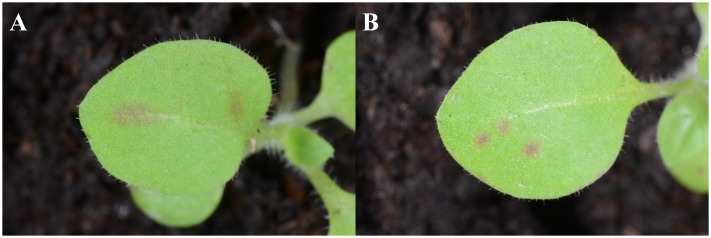
**Visual monitoring of the PVY-Ros1 aphid transmission process**. Details of representative tobacco plants inoculated with PVY-Ros1 **(A)** using viruliferous aphids or **(B)** mechanically. Aphid transmission frequently produced elongated red infection foci suggesting inoculation in vascular bundles. Pictures were taken at 5 dpi.

Most plant viruses are transmitted by vectors. In the particular case of potyviruses, transmission by aphids is a distinctive trait of the genus. We wondered whether the Rosea1 marker would allow the visual monitoring of the aphid transmission process. To this end, and after a standard starvation period, aphid populations were allowed to feed on red-colored PVY-Ros1-infected tobacco tissue for 10 min to acquire the virus. Next they were individually transferred at different densities to healthy tobacco test plantlets and confined there for overnight inoculation. Some plantlets were excluded from aphid inoculation to serve as controls, and a subgroup of them was mechanically inoculated (positive controls). Aphids were finally eliminated by insecticide treatment. Unlike the control plants into which no aphids were released, red spots started to appear 5 days later in some of the plants had come into contact with the viruliferous aphids (**Figure 5A**), and at the same time as in the mechanically inoculated controls (**Figure 5B**). Later, all these plants exhibited infection symptoms on their upper non-inoculated leaves, as well as red coloration. **Table 1** summarizes the results of eleven independent experiments to compare the virus transmission rate at three different aphid densities. In all cases, viral infection was easily monitored by the appearance of a red coloration induced by the Rosea1 marker. The aphid-mediated PVY-Ros1 transmission rate ranged from 84% at a vector density of 10 aphids per plant to 23% at 1 aphid per plant (**Table 1**). All the plants with red infection foci on their inoculated leaves later developed systemic infection. Once again, the ease of monitoring aphid-mediated virus transmission makes PVY-Ros1 an excellent tool to quantify the effect of phytosanitary treatments on disease transmission by viruliferous vectors. Incidentally we observed that compared to the mechanically inoculated plants in which the vast majority of infection foci were circular (e.g., see **Figure 2B**), the plants inoculated by aphids showed a large proportion of irregularly shaped and elongated foci that developed along leaf nerves (**Figure 5A**). This observation suggests that the epidermal cells from the leaf surface are initially infected during mechanical inoculation, while aphid-mediated inoculation may more frequently initiate infection in vascular tissue. Tracking the initial stages of virus infection by insect vectors was recently reported for a transmissible variant of *Cucumber mosaic virus* tagged with GFP (Krenz et al., 2015). Our results indicate that PVY-Ros1 can be used to learn about the inoculation sites by aphids in potyviruses.

**Table 1:** Aphid transmission of PVY-Ros1.

		Transmission		
Number of aphids	Number of repetitions	Total^1^	Percentage	Range^2^
10	4	38/45	84.4%	66.7–100
5	3	14/32	43.8%	16.7–62.5
1	4	11/48	22.9%	0–33.3

^1^*Number of infected plants over number of test plants. Data combined from the number of repeated experiments as indicated. ^2^Range of transmission rates for each individual repetition of the experiment is indicated. When corrected by number of aphids (transmission per aphid), rates did not significantly differ at the 0.05 level according to the Tukey (HSD) test*.

### PVY-Ros1 as a Molecular Farming Tool to Produce Anthocyanins in Biofactory Plants

**FIGURE 6:**
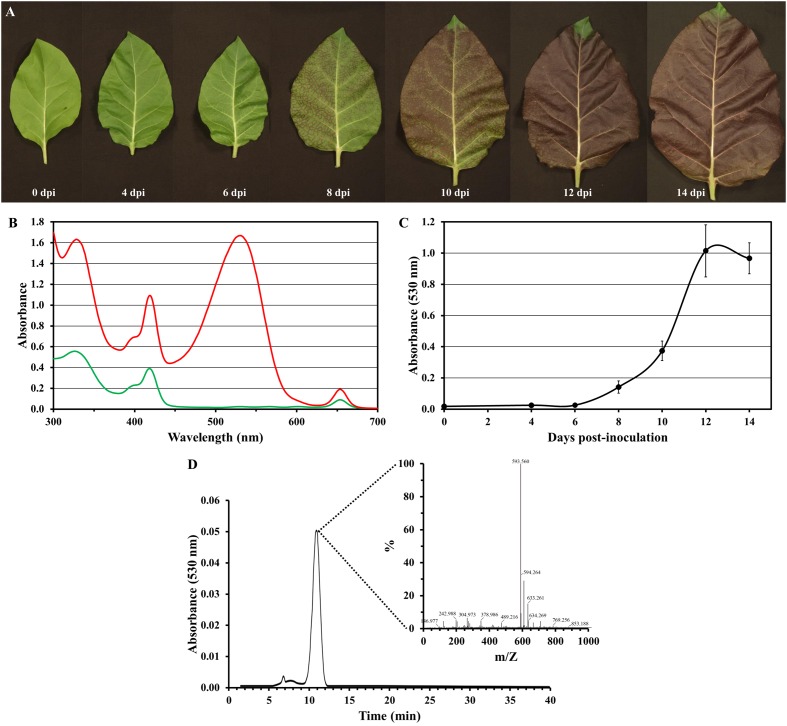
***Potato virus Y*-Ros1-mediated anthocyanin production in tobacco plants**. **(A)** Representative upper non-inoculated leaves sampled from plants mechanically inoculated with PVY-Ros1. Pictures were taken at different dpi, as indicated. **(B)** Absorption spectra of the methanol extracts obtained from lower non-symptomatic (green line) and upper symptomatic (red line) tissue from a PVY-Ros1-infected tobacco plant (12 dpi). **(C)** Plot of average absorbance at 530 nm versus time post-inoculation of the methanol extracts from the upper non-inoculated tissues of the tobacco plants infected with PVY-Ros1. Error bars represent the standard error median. **(D)** Identification of cyanidin-3-O-rutinoside in the extracts of PVY-Ros1 infected tobacco tissue by a HPLC separation coupled to MS analysis.

**FIGURE 7:**
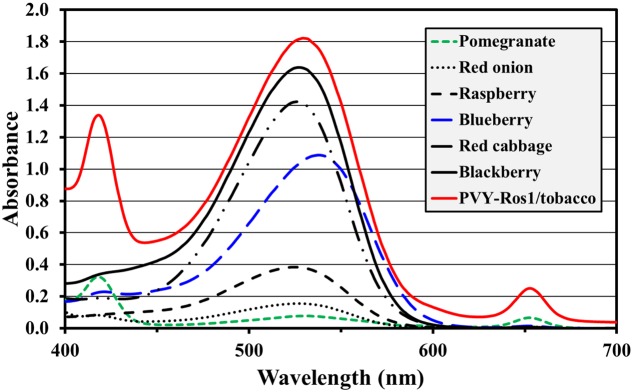
**Comparison of anthocyanin accumulation in PVY-Ros1-infected tissue with different fruits and vegetables**. Visible spectra (400–700 nm) of the extracts obtained with acidified methanol (ratio 100 ml per 1 g fresh weight) from PVY-Ros1-infected tobacco leaves (red line) and different fruits and vegetables, as indicated.

Anthocyanins are water-soluble natural pigments produced in plants, which have been paid close attention thanks to their health-promoting properties (Zhang et al., 2014; Passeri et al., 2016). While performing the previously described experiments, we were astonished by the red pigmentation intensity induced by PVY-Ros1, particularly in tobacco plants (e.g., see **Figure 2C**). We envisioned that PVY-Ros1 could also be used in molecular farming to very quickly induce large accumulations of anthocyanins in biofactory plants. With this idea in mind, we studied the dynamics of PVY-Ros1-induced anthocyanin accumulation in tobacco plants, identified the main anthocyanin produced in the infected tissues of tobacco and estimated anthocyanin yields. For this purpose, we mechanically inoculated a series of 5-week-old tobacco plants with PVY-Ros1 in one leaf and harvested the upper non-inoculated tissue at different dpi (**Figure 6A**). From these tissues, anthocyanins were extracted in acidified methanol and quantified by a spectrophotometric analysis. **Figure 6B** compares the ultraviolet-visible absorption spectra of two extracts obtained from the same plant. The green extract (green line in spectra) was obtained from lower non-symptomatic tissue and the dark red extract (red line) from upper symptomatic tissue. An intense absorption band centered at 530 nm indicated the vast accumulation of anthocyanins in the latter tissue. An analysis of the absorbance in extracts from the tissue collected at different dpi showed that substantial amounts of anthocyanins started to accumulate in infected tissues at around 8 dpi and reached the maximum accumulation at around 12 dpi (**Figure 6C**). A high performance liquid chromatography (HPLC), coupled to mass spectrometry (MS), analysis of an extract that corresponded to 12 dpi indicated that cyanidin-3-O-rutinoside (antirrhinin) was the main anthocyanin present in the extract (**Figure 6D**). When considering the molar extinction coefficient of this species (26,900 M^-1^cm^-1^), we estimated that accumulation was around 275 mg of anthocyanin per 100 g of tobacco fresh tissue. This amount is similar to those that accumulate in fruits and other plant tissues distinguished for their high anthocyanin content (Wu et al., 2006; Zhao et al., 2013). In order to avoid unnecessary speculations, we compared the anthocyanin content of the PVY-Ros1-infected tobacco tissue (12 dpi) with some seasonal fruits and vegetables that we purchased in a local market. Extracts were obtained under the same conditions using acidified methanol and anthocyanins were quantified by a spectrophotometric analysis. Interestingly, this analysis indicated that the anthocyanin accumulation in the PVY-Ros1-infected tobacco tissue was greater than that in blackberries, red cabbage, blueberries, raspberries, red onion or pomegranate (**Figure 7**).

While the PVY-Ros1/tobacco system produces large amounts of natural anthocyanin cyanidin-3-O-rutinoside, the co-expression or silencing of some particular enzymes of the anthocyanin biosynthetic pathway or vacuole transporters may allow the efficient production of novel anthocyanins with a particular commercial value. These enzymes may be expressed using the same virus or other viral vectors, e.g., those that derive from *Tobacco mosaic virus* or *Potato virus X*, which produce compatible infections with PVY. Potyvirus-derived vectors can co-express several proteins whose cDNAs are inserted into different virus genome positions (Kelloniemi et al., 2008), or into a single expression cassette (Bedoya et al., 2010; Majer et al., 2017).

Substantial amounts of anthocyanins have also been reported in transgenic tobacco plants that express sweet potato (*Ipomoea batatas* [L.] Lam) transcription factor IbMYB1a. The greatest accumulation, that of 110 mg per 100 g fresh tissue, has been reported in the leaves of a line in which this transcription factor was expressed under the control of the sporamin promoter (An et al., 2015). This means that the virus-based strategy described herein produced a larger amount of anthocyanins (about 2.5-fold) by a much less complicated process than plant stable transformation. Moreover, our virus vector strategy used adult plants in which developmental problems induced by high anthocyanin accumulation were avoided. In this way, using plant virus-derived systems to express the enzymes or regulatory factors involved in the biosynthesis of valuable compounds may add a series of advantages compared to transgenic plants. First, viral genomes can be quickly and easily manipulated. Second, the production of compounds of interest can be triggered in adult plants. Third, products of interest can be harvested only a few days after inoculation. We previously showed the production of lycopene and other carotenoids in tobacco tissues using a TEV-derived vector that expresses biosynthetic enzymes of bacterial origin (Majer et al., 2017). Unfortunately, unlike stable transformation strategies, the amount of genetic information that can be expressed using plant viruses is limited. We envision that the combined use of recombinant plant viruses and genetically transformed host plants will extend the possibilities of producing valuable compounds in increasingly more sophisticated molecular farming approaches.

### PVY-Ros1 Is a Versatile Biotechnological Tool that Serves Multiple Purposes

In conclusion, in this work we built an infectious recombinant PVY clone that efficiently expresses the *A. majus* MYB-type Rosea1 transcription factor, which strongly activates anthocyanin accumulation in infected tissues. Whereas the plasmid in which the recombinant virus was contained (pGPVY-Ros1) was rather unstable when amplified in *E. coli* or *A. tumefaciens*, and was not easy to deal with, the recombinant virus (PVY-Ros1) generated in a host plant was highly infectious and stable. By using PVY-Ros1 as an inoculum, we demonstrated how infection foci and infected plants can be easily quantified by the naked eye without having to resort to molecular analyses or complex instrumentation. Our experimental results also showed how PVY-Ros1 facilitated the analyses of phytosanitary products, such as silver nanoparticles, or allowed the monitoring of virus aphid transmission, an essential process for disease dissemination. Finally, we also demonstrated how PVY-Ros1 can be used in molecular farming to trigger the production of large amounts of antioxidant anthocyanins (275 mg per 100 g of fresh weight) in biofactory plants in only 12 days.

## Author Contributions

J-AD conceived the project and designed the experiments with inputs form all the other authors. TC, MM, J-JL-M, and J-AD performed the experiments and analyzed the data. J-AD wrote the manuscript with inputs from all the other authors. All authors read and approved the final manuscript.

## Conflict of Interest Statement

The authors declare that the research was conducted in the absence of any commercial or financial relationships that could be construed as a potential conflict of interest.
