# Supplementary material for: A Recombinant Potato virus Y Infectious Clone Tagged with the Rosea1 Visual Marker (PVY-Ros1) Facilitates the Analysis of Viral Infectivity and Allows the Production of Large Amounts of Anthocyanins in Plants
Source: Front Microbiol. 2017 Apr 6;8:611. doi: 10.3389/fmicb.2017.00611 (PMC5382215; doi:10.3389/fmicb.2017.00611)
Supplement: Supplementary file 1 [file Table_1.PDF]

TABLE S1. Oligodeoxynucleotide primers used to build and analyze PVY-Ros1.

Name	Sequence (5'-3')[1]	Orientation[2]	Use
PI	GTTGGCAAACGCCTAAAGAT	R	Reverse transcription of the three genome fragments
PII	ACCTTTAATTGGCAGAACGC	R	
PIII	TTTTGTCTCCTGATTGAAG	R	
PIV	GGCGGGTCTCGGAGGAAATTAAAACAACTCAATACAAC	F	PCR amplification of the 5' genome fragment
PV	CCGCGGTCTCCTTATTAAATCGCTCGCTCAATC	R	
PVI	GGCGGGTCTCGATAATTATTTCAATACTAAGTG	F	PCR amplification of the central genome fragment
PVII	CCGCGGTCTCCTTTCAGCCTACAAACTGTTTGG	R	
PVIII	GGCGGGTCTCGGAAAGTATCTGTTGAATATGGG	F	PCR amplification of the 3' genome fragment
PIX	CCGCGGTCTCCTTTTGTCTCCTGATTGAAGTTTACAG	R	
PX	CCATGCACGGTACATTCAGG	F	PCR to mutagenize the endogenous Eco31I recognition site in the central genome fragment (silent mutation)
PXI	CTGATTGAAGTTTACAGTCA	R	
PXII	CCGCGGTCTCCTTGATGGTATACTTCATAAG	R	PCR to split between NIb and CP in the third genome fragment
PXIII	GGCGGGTCTCGGCAAATGACACAATTGATGC	F	
PXIV	GGCGGGTCTCGTCAAGCAAACGACATGGAAAAGAATTGTCGTGG	F	PCR amplification of Rosea1 flanked with the sequences to complement NIb/CP proteolytic cleavage
PXV	CCGCGGTCTCCTTGCCTGATGATATACCTCATATGAGTCATTTCCAATTTGTTGGGCC	R	
PXVI	GCAAATGACACAATTGATGC	F	RT-PCR diagnosis of PVY
PXVII	CATGTTCTTGACTCCAAGTAG	R	

[1]Eco31I recognition and cleavage sequences are on a gray background and underlined, respectively.
[2]Primer orientation in relation to the PVY RNA genome: forward (F) or reverse (R).
