# Supplementary material for: A Recombinant Potato virus Y Infectious Clone Tagged with the Rosea1 Visual Marker (PVY-Ros1) Facilitates the Analysis of Viral Infectivity and Allows the Production of Large Amounts of Anthocyanins in Plants
Source: Front Microbiol. 2017 Apr 6;8:611. doi: 10.3389/fmicb.2017.00611 (PMC5382215; doi:10.3389/fmicb.2017.00611)
Supplement: Supplementary file 2 [file Table_2.PDF]

TABLE S2. Effect of silver nanoparticles on PVY-Ros1-induced infection foci on tobacco leaves.

Plant	Leaf[1]	Number of infection foci		
		Mock	100 ppm	1000 ppm
1	4	322	104	5
	5	198	142	20
2	4	227	159	2
	5	181	150	20
3	4	202	119	30
	5	218	53	35
4	4	138	100	26
	5	207	45	16
5	4	182	40	16
	5	188	49	33
6	4	316	25	46
	5	422	55	20

[1]In each plant, true leaves 4 and 5 were mechanically inoculated with PVY-Ros1.
