# Supplementary material for: A Recombinant Potato virus Y Infectious Clone Tagged with the Rosea1 Visual Marker (PVY-Ros1) Facilitates the Analysis of Viral Infectivity and Allows the Production of Large Amounts of Anthocyanins in Plants
Source: Front Microbiol. 2017 Apr 6;8:611. doi: 10.3389/fmicb.2017.00611 (PMC5382215; doi:10.3389/fmicb.2017.00611)
Supplement: Supplementary file 3 [file Data_Sheet_1.PDF]

DATASET S1. Sequence of recombinant PVY-Ros1 clone (GenBank accession number KY780083).

The start and stop codons of the main open reading frame are in green and dark red, respectively. Limits between cistrons are marked on blue background. *Rosea1* cDNA from *Antirrhinum majus* is in red and flanking sequences inserted to complement the split NIb/CP cleavage site are in blue. Mutations with respect to HM367076 are underlined.

AAATTAAAACAACTCAATACAACATAAGAAAAACAGCGCAAAAACACTCATAAACGCTTATTCTCACTCAAGCAT
CTTGCTAAGTTTCAGTTCAAATCATTTCCTTGCAATTCTCTTAAACAATATTGGAAACCATTTCAACTCAACAAG
CAATCTCATCACTTCCAACCAACTTGAAATCCTCG**ATG**GCAACTTACATGTCAACGATCTGTTTCGGTTCGTTTG
AATGCAAGCTACCATACTCACCCGCCTCTTGCGGGCTTATTGTGAAGGAACGAGAAGTGCTGGCTTCCGTTAATC
CTTTCGCAGATCTGGAAACACAACTTAGTGCACGATTGCTCAAGCAAGAATATGCTACTGTTCGTGTGCTCAAGA
ACGGTACTTTTACGTATCGATACAAGACTGATGCCCAGATAAAGCGCATTCAGGAGAAACTGGAGAGGAAGGATA
GGGAAGAATATCACTTCCAAATGGCCGCTCCTAGTATTGTGTCAAAGATTACCATAGCTGGTGGAGATCCTCCAT
CAAAGTCTGAGCCACAAGCACCAAGAGGGATCATTCATACAACTCCAAGGGTGCGTAAAGTCAAGACACGCCCCA
TAATAAAGTTGACAGAAGGCCAGATGAATCACCTCATTAAGCAGGTAAAGCAGATTATGTCGGAGAAGAGAGGGT
CTGTCCACTTAATTAATAAGAAGACCACTCATGTTCAATATAAGGAGATACTTGGTGCAACTCGCGCAGCGGTTC
GAACTGCACATATGATGGGTTTGCGACGGAGAGTGGACTTCCGATGTGATATGTGGACAGTTGGACTTTTGCAAC
GTCTCGCTCGGACGGACAAATGGTCCAATCAAGTCCGCACTATCAACATACGAAGGGGTGATAGTGGAGTCATCT
TGAACACAAAAAGCCTCAAAGGCCACTTTGGTAGAAGTTCAGGAGACTTGTTCATAGTGCGCGGATCACATGAAG
GGAAATTGTACGATGCACGATCTAGAGTTACTCAGAGTGTTTTGGACTCAATGATCCAGTTTTCGAATGCTGATA
ATTTTTGGAAGGGTCTGGACGGTAATTGGGCACGAATGAGATATCCTTCGGATCACACATGTGTAGCTGGTTTAC
CTGTCGAAGATTGTGGTAGGGTTGCTGCATTGATGGCACACAGTATCCTCCCGTGCTATAAGATAACCTGCCCCA
CCTGTGCTCAACAGTATGCCAGCTTGCCGGTTAGCGATCTGTTTAAGCTATTGCATAAACATGCAAGAGATGGTT
TGAACCGATTGGGAGCAGATAAAGACCGGTTTATACATGTTAATAAGTTCTTGATAGCGTTAGAGCATCTAACTG
AACCGGTGGATTTGAACCTCGAGCTTTTCAATGAGATATTTAAATCCATAGGGGAGAAGCAGCAAGCACCGTTCA
AGAATTTAAATGTCTTAAATAATTTCTTCCTGAAAGGAAAAGAAAATACAGCTCATGAATGGCAGGTGGCTCAAT
TGAGTTTGCTCGAATTAGCAAGGTTCCAGAAGAATAGAACTGATAACATCAAGAAGGGTGATATATCTTTCTTCA
GAAATAAATTATCTGCCAAGGCAAATTGGAATCTGTATTTGTCGTGCGACAACCAATTGGACAAAAATGCAAATT
TCCTGTGGGGACAAAGGGAGTATCATGCTAAGCGGTTTTTCTCAAATTTCTTTGAGGAAATTGATCCAGCAAAGG
GATACTCAGCATATGAAATCCGCAAGCATCCAAATGGAACAAGGAAGCTTTCAATTGGTAACTTAGTTGTCCCAC
TTGATTTAGCTGAGTTTAGGCAGAAGATGAAAGGTGACTATAGGAAGCAACCAGGGGTCAGCAGAAAGTGCACGA
GTTCGAAAGATGGTAATTATGTGTATCCCTGTTGTTGCACAACACTTGATGATGGTTCGGCCATTGAATCAACAT
TCTATCCACCAACTAAAAAGCACCTTGTAATAGGCAATAGTGGTGACCAAAAGTTTGTTGATTTACCAAAAGGGG
ATTCAGAGATGTTATACATTGCCAAGCAGGGTTATTGTTATATTAACGTGTTTCTTGCAATGCTAATTAACATTA
GCGAGGAGGATGCAAAGGATTTCACAAAGAAAGTCCGCGACATGTGTGTGCCGAAGCTTGGAACCTGGCCAACTA
TGATGGATTTGGCGACCACTTGTGCTCAAATGAGAATATTCTATCCTGACGTGCATGATGCAGAGCTGCCTAGAA
TATTGGTTGACCATGACACTCAAACGTGTCATGTGGTTGACTCATTTGGCTCGCAGACAACTGGATATCATATTC
TAAAAGCATCCAGCGTGTCTCAACTTATCTTGTTTGCAAATGATGAATTAGAATCTGATATAAAACATTATAGAG
TTGGTGGCGTTCCTAATGCATGCCCTGAACTTGGGTCCACGATATCACCTTTTAGAGAAGGAGGAGTTATAATGT
CTGAGTCGGCAGCGCTGAAACTGCTTTTGAAGGGAATTTTTAGACCTAAGGTGATGAGACAGTTGCTGTTAGATG
AGCCTTACCTGTTGATTCTATCAATATTATCCCCTGGCATACTGATGGCTATGTATAATAATGGGATTTTTGAAC
TTGCGGTAAGATTGTGGATTAATGAGAAACAATCCATAGCTATGATAGCATCGCTACTATCAGCTTTAGCCCTAC
GAGTGTCAGCGGCAGAAACACTCGTCGCACAGAGAATTATAATTGATGCTGCAGCTACAGACCTCCTTGATGCTA
CGTGTGATGGATTCAACCTACATCTAACGTACCCCACTGCATTAATGGTATTACAAGTTGTTAAGAATAGAAATG
AATGTGATGATACCCTATTCAAGGCGGGTTTTCCAAGTTACAACACGAGTGTTGTGCAGATTATGGAAAAAAATT
ATCTAAGTCTCTTGGACGATGCTTGGAAAGATTTAACTTGGCGGGAAAAATTATCCGCAACATGGTACTCATACA
GAGCAAAACGCTCTATCACTCGGTACATAAAACCCACAGGAAGGGCAGATTTGAAAGGGTTATACAACATATCAC
CACAAGCATTCTTGGGCCGAAGCGCCCAGGTGGTCAAAGGCACTGCCTCAGGATTGAGCGAGCGATTTAATAATT
ATTTCAATACTAAGTGTGTAAATATTTCATCCTTTTTCATTCGTAGAATCTTTAGGCGTTTGCCAACTTTCGTCA
CTTTTGTTAACTCATTATTAGTTATTAGTATGTTAACTAGTGTAGTGGCAGTGTGTCAGGCAATAATTTTAGATC
AGAGGAAGTATAGGAGAGAAATCGAGTTGATGCAGATAGAGAAGAATGAGATTGTCTGCATGGAGCTATATGCAA
GTTTACAGCGCAAACTTGAACGCGATTTCACATGGGATGAGTACATTGAGTATTTGAAGTCAGTAAACCCTCAGA
TAGTTCAGTTTGCTCAAGCGCAGATGGAAGAATATGATGTGCGACACCAGCGTTCCACACCAGGTGTTAAAAATT
TGGAACAAGTGGTAGCATTTATGGCTTTAGTCATCATGGTGTTCGATGCTGAAAGGAGTGATTGCGTTTTCAAAA
CTCTCAATAAATTTAAGGGTGTCCTTTCCTCGCTGGACCATGAAGTTCGACATCAGTCCTTAGACGATGTGATCA
AGAATTTTGATGAGAGGAATGAGATTATTGATTTTGAGTTGAGTGAGGACACAATTCGAACATCATCAGTGCTAG
ATACAAAGTTTAGTGATTGGTGGGACCGACAAATCCAGATGGGACATACACTTCCACATTACAGAACCGAGGGGC
ACTTCATAGAATTTACAAGAGCAACTGCTGTTCAAGTGGCTAATGACATTGCCCATAGTGAACACCTAGACTTTT
TAGTAAGGGGAGCTGTTGGGTCTGGAAAGTCAACTGGGTTGCCTGTTCATCTTAGCGTAGCCGGATCTGTGCTTT

TAATTGAACCAACGCGACCACTGGCGGAGAACGTTTTCAAACAGCTATCTAGTGAACCATTCTTCAAGAAGCCAA
CACTGCGTATGCGCGGAAATAGTATATTTGGCTCTTCTCCAATCTCCGTCATGACTAGCGGATTCGCGCTACACT
ACTTCGCCAATAATCGCTCCCAATTAGCTCAGTTCAACTTTGTAATATTTGATGAGTGCCATGTTCTGGATCCTT
CCGCAATGGCGTTCCGCAGTCTGCTGAGTGTTTATCATCAAGCATGCAAAGTATTAAAAGTGTCAGCTACTCCAG
TGGGAAGGGAGGTTGAATTCACAACACAGCAGCCAGTCAAGTTAATAGTGGAGGACACACTGTCTTTCCAATCAT
TTGTTGATGCACAAGGTTCTAAAACTAATGCTGATGTTGTTCAGTTTGGTTCAAACGTACTTGTGTATGTGTCGA
GCTACAATGAAGTTGATACCTTGGCTAAGCTCCTAACAGACAAGAATATGATGGTCACAAAGGTTGATGGCAGAA
CAATGAAGCACGGTTGCCTAGAAATTGTCACAAAAGGAACCAGTGCGAGGCCACATTTTGTTGTAGCAACCAACA
TAATTGAAAATGGAGTGACTTTGGACATAGACGCGGTTGTGGATTTTGGGTTGAAAGTCTCACCATTCTTGGACA
TTGACAATAGGAGCATTGCCTACAATAAGGTGAGTATTAGCTATGGTGAAAGAATTCAAAGGTTGGGTCGTGTTG
GACGCTTCAAGAAAGGAGTAGCATTGCGCATTGGACACACTGAGAAGGGAATTATTGAAATTCCAAGCATGGTTG
CTACTGAGGCGGCTCTTGCTTGCTTTGCATATAACTTGCCAGTGATGACAGGCGGCGTCTCAACTAGTCTGATTG
GCAATTGTACTGTGCGCCAGGTTAAAACAATACAGCAATTTGAATTGAGTCCATTCTTTATCCAGAATTTCGTTG
CTCATGATGGATCAATGCATCCTGTCATACATGACATTCTTAAAAAGTATAAACTGCGAGATTGTATGACACCTT
TGTGCGATCAGTCTATACCATACAGGGCATCGAGCACTTGGTTATCGGTTAGTGAATATGAGCGACTTGGAGTGG
CCTTAGAAATTCCAAAGCAAGTCAAAATTGCATTCCATATCAAAGAGATCCCTCCTAAGCTCCACGAAATGCTTT
GGGAAACGGTTGTCAAATACAAAGACGTTTGCTTATTTCCAAGCATTCGAGCATCGTCCATCAGCAAAATCGCAT
ACACATTGCGTACAGACCTCTTCGCCATCCCAAGGACTCTAATATTGGTGGAGAGATTACTTGAAGAGGAGCGAG
TGAAGCAGAGCCAATTCAGAAGTCTCATCGATGAAGGGTGCTCAAGTATGTTTTCAATTGTTAACTTGACCAACA
CTCTCAGAGCCAGATATGCAAAAGATTACACCGCAGAGAACATACAAAAACTTGAGAAAGTGAGAAGTCAATTGA
AAGAATTCTCAAATCTGGATGGTTCTGCATGTGAGGAGAATTTAATAAAGAGGTATGAGTCTTTGCAGTTCGTTC
ATCACCAAGCTGCAACGTCACTTGCAAAGGATCTCAAGTTGAAGGGGACCTGGAAGAAATCATTAGTGGCTAAAG
ACTTGATCATAGCAGGCGCTGTTGCAATTGGTGGTATAGGACTCATATATAGTTGGTTCACACAATCAGTTGAGA
CTGTGTCCCATCAAGGGAAAAATAAATCCAAAAGAATCCAAGCCTTGAAGTTTCGCCATGCTCGTGACAAAAGGG
CTGGCTTTGAAATTGACAGCAATGATGACACAATAGAGGAATTCTTTGGATCTGCATATAGGAAAAAGGGAAAAG
GTAAAGGTACCACAGTCGGTATGGGCAAGTCAAGCAGGAAGTTCATCAACATGTATGGGTTTGATCCAACAGAGT
ACTCATTCATCCAGTTCGTTGATCCACTCACTGGGGCGCAAATAGAAGAGAATGTCTATGCTGACATTAGAGACA
TTCAAGATAGATTTAGTGAAGTGCGAAAGAAAATGGTTGAGAATGATGACATTGAAATGCAAGCCTTGAGTAGTA
ACACAACCATACATGCATACTTCAGGAAAGATTGGTCTGACAAAGCTTTGAAGATTGACTTAATGCCACACAACC
CACTCAAAGTCTGTGACAAAACAAATGGCATTGCAAAATTTCCTGAGAGAGAGCTCGAACTAAGGCAGACTGGAC
CAGCTGTAGAAGTCAATGTGCAGGACATACCAGCACAGGAGGTGGAGCATGAAGCTAAATCGCTCATGAGAGGTT
TGAGAGACTTCAACCCAATCGCCCAAACAGTTTGTAGGCTGAAAGTATCTGTTGAATATGGGACATCAGAGATGT
ACGGTTTTGGATTTGGAGCATACATAATAGCGAACCACCATTTGTTTAGGAGTTACAATGGTTCCATGGAGGTGC
GATCCATGCACGGTACATTCAGGGTGAAGAATCTACACAGTTTGAGCGTTCTGCCAATCAAAGGTAGGGACATAA
TCCTCATAAAAATGCCGAAGGATTTCCCTGTCTTTCCACAGAAATTGCATTTCCGAGCTCCTATACAGAATGAAA
GAGTTTGTTTAGTTGGGACCAACTTTCAGGAGAAGTATGCATCGTCAATCATCACAGAAACAAGCACTACTTACA
ATATACCAGGTAGCACATTCTGGAAGCATTGGATTGAAACAGACAATGGACATTGTGGACTACCAGTGGTAAGCA
CTGCCGATGGATGTCTAGTCGGAATTCACAGTTTGGCAAACAATGCACACACCACGAATTACTACTCAGCCTTCG
ATGAGGATTTTGAAAGCAAGTACCTCCGGACCAATGAGCACAATGAATGGGTCAAGTCTTGGAAATATAATCCAG
ACACAGTGTTGTGGGGCCCGTTGAAACTTAAAGACAGCACTCCCAAAGGGTTATTTAAAACAACAAAGCTTGTGC
AAGATCTAATCGAGCATGATGTAGTGGTGGAGCAAGCTAAGCACTCTGCGTGGATGTTTGAAGCCTTGACAGGAA
ATTTGCAAGCTGTCGCAACAATGAAGAGCCAATTAGTAACCAAGCATGTAGTTAAAGGAGAGTGTCGACACTTCA
AGGAGTTCCTGACTGTGGATGCAGAAGCAGAGGCATTCTTCAGGCCTTTGATGGATGCGTATGGAAAAGCTTGC
TGAACAGAGATGCGTACATCAAGGACATAATGAAGTATTCAAAACCTATAGATGTTGGTATCGTGAATTGTGATG
CATTCGAGGAAGCCATCAATAGGGTTATCATCTACCTGCAAGTGCACGGCTTCCAGAAGTGTACATATGTCACTG
ACGAGCAAGAAATTTTCAAAGCGCTTAACATGAAAGCTGCAGTCGGAGCCATGTATGGTGGCAAAAAGAAAGACT
ATTTTGAGCATTTCACTGATGCAGACAAGGAAGAAATAGTCATGCAAAGCTGTCTGCGATTGTATAAAGGCTTGC
TCGGCATTTGGAATGGATCATTGAAGGCAGAGCTCCGGTGCAAGGAAAAGATACTTGCAAATAAGACGAGGACAT
TCACTGCTGCACCTCTAGACACTTTGCTGGGTGGTAAAGTGTGTGTTGACGACTTCAATAATCAATTTTATTCAA
AGAATATTGAGTGCTGTTGGACAGTTGGGATGACTAAGTTTTATGGTGGTTGGGATAAACTGCTTCGGCGTTTAC
CTGAGAATTGGGTATACTGTGATGCTGATGGCTCACAGTTTGATAGTTCACTAACTCCATACTTAATCAATGCTG
TTCTCACCATCAGAAGCACATACATGGAAGACTGGGATGTGGGGCTGCAAATGCTGCGTAATTTATACACTGAGA
TTGTTTACACACCTATTTCAACTCCAGATGGAACAATTGTTAAGAAGTTCAGAGGAAATAACAGTGGTCAGCCTT
CTACTGTTGTGGACAACTCTCTTATGGTCGTCCTTGCCATGCACTATGCTTTCATCAGAGAAGGCATTGAGTTTG
AAGAAACTGACAGCACGTGCGTGTTCTTTGTTAATGGTGATGATTTGCTGATTGCTGTGAATCCGGATAAAGAGG
ACATTCTTGACAGATTGTCACAACACTTCTCAGATCTTGGCTTAAATTATGATTTCTCGTCAAGAACAAGAAATA
AGGAAGAGTTGTGGTTTATGTCTCATAGGGGCCTACTGATTGAGGGCATGTACGTGCCGAAACTTGAAGAAGAAA
GGATTGTGTCCATTCTCCAATGGGACAGAGCAGACTTGGCTGAACACAGGCTTGAGGCGATTTGCGCAGCTATGA
TAGAGTCCTGGGGTTATTCTGAACTAACACACCAAATCAGGAGATTCTACTCATGGTTATTGCAACAGCAACCTT
TTGCAACAATAGCGCAGGAGGGGAAGGCTCCTTATATAGCAAGCATGGCATTAAGGAAACTGTATATGGATAGGG
CTGTGGATGAGGAAGAGCTTAGAGCCTTCACTGAAATGATGGTCGCATTAGACGATGAGTTTGAGTTTGACTCTT

ATGAAGTATACCATCA**AGCAAACGACATGGAAAAGAATTGTCGTGGAGTGAGAAAAGGTACTTGGACCAAAGAAG**
AAGACACTCTCTTGAGGCAATGTATAGAAGAGTATGGTGAAGGGAAATGGCATCAAGTTCCACACAGAGCAGGGT
TGAACCGGTGTAGGAAGAGTTGCAGGCTGAGGTGGTTGAATTATCTGAGGCCAAATATCAAAAGAGGTCGGTTTT
CGAGAGATGAAGTGGACCTAATTGTGAGGCTTCATAAGCTGTTGGGTAACAAATGGTCGCTGATTGCTGGTAGAA
TTCCTGGAAGGACAGCTAATGACGTGAAGAACTTTTGGAATACTCATGTGGGGAAGAATTTAGGCGAGGATGGAG
AACGATGCCGGAAAAATGTTATGAACACAAAAACCATTAAGCTGACTAATATCGTAAGACCCCGAGCTCGGACCT
TCACCGGATTGCACGTTACTTGGCCGAGAGAAGTCGGAAAAACCGATGAATTTTCAAATGTCCGGTTAACAACTG
ATGAGATTCCAGATTGTGAGAAGCAAACGCAATTTTACAATGATGTTGCGTCGCCACAAGATGAAGTTGAAGACT
GCATTCAGTGGTGGAGTAAGTTGCTAGAAACAACGGAGGATGGGGAATTAGGAAACCTATTCGAGGAGGCCCAAC
AAATTGGAAATGACTCATATGAGGTATATCATCAGGCAAATGACACAATTGATGCAGGAGGAAGCAACAAGAAAG
ATACAAAACCAGAGCAAAGCAGCATCCAGTCAAACCCGAACAAAGGAAAAGATAAAGATGTGAATGCCGGCACAT
CTGGGACACACACTGTACCGAGAATCAAGGCTATCACGTCCAAAATGAGAATGCCCAAAAGCAAGGGAGCAGCTG
TGCTGAATTTAGAACACTTGCTTGAGTATGCTCCACAACAAATTGATATTTCAAATACTCGGGCAACTCAATCAC
AGTTTGATACGTGGTATGAAGCAGTGCGGATGGCATACGACATAGGAGAAACTGAGATGCCAACTGTGATGAATG
GGCTTATGGTTTGGTGCATTGAAAATGGAACCTCGCCAAATGTCAACGGAGTTTGGGTTATGATGGATGGGAATG
AACAAGTTGAGTACCCGTTGAAACCAATCGTTGAGAATGCAAAACCAACCCTTAGGCAAATCATGGCACATTTCT
CAGATGTTGCAGAAGCGTATATAGAAATGCGCAACAAAAAGGAACCATATATGCCACGATATGGTTTAATTCGAA
ATCTGCGGGATATGGGTTTAGCGCGTTATGCTTTTGACTTTTATGAGGTCACATCACGAACACCAGTGAGGGCTA
GGGAAGCGCAAATTCAAATGAAGGCCGCAGCATTGAAATCAGCTCAACCTCGACTTTTCGGGTTGGACGGTGGCA
TCAGTACACAAGAGGAGAACACAGAGAGGCACACCACCGAGGATGTCTCTCCAAGTATGCATACTCTACTTGGAG
TCAAGAACATG**TGA**TGTAGTGTCTCTCCGGACGATATATAAATATTTACATATGCAGTAAGTATTTTGGCTTTTC
CTGTACTACTTTTATCATAATTAATAATCAGTTTGAATATTACTAATAGATAGAGGTGGCAGGGTGATTTCGTCA
TTGTGGTGACTCTATCTGTTAATTTCGCATTATTAAGTCTTAGATAAAAGTGCCGGGTTGTCGTTGTTGTGGATG
ATTCATCGATTAGGTGATGTTGCGATTCTGTCGTAGCAGTGACTATGTCTGGATCTATCTGCTTGGGTGGTGTTG
TGATTTTGTCATAACAGTGACTGTAAACTTCAATCAGGAGAC
